# HS-GC-IMS with sensory evaluation technique to analyze volatile flavor compounds of jujube flowers

**DOI:** 10.3389/fpls.2025.1590072

**Published:** 2025-09-12

**Authors:** Peixing Ren, Ruirui Dao, Lili Wang, Noor Muhammad, Yaxin Sang, Mengjun Liu, Zhihui Zhao

**Affiliations:** ^1^ College of Horticulture, Hebei Agricultural University, Baoding, Hebei, China; ^2^ Research Center of Chinese Jujube, Hebei Agricultural University, Baoding, Hebei, China; ^3^ College of Food Science and Technology, Hebei Agricultural University, Baoding, Hebei, China

**Keywords:** jujube flowers, different periods, varieties, GC-IMS, PCA

## Abstract

The combination of headspace gas chromatography-ion mobility spectrometry (HS-GC-IMS) and sensory evaluation technique was employed to detect and analyze the volatile organic compounds (VOCs) in jujube flowers at different developmental stages across various varieties, and to compare them with other plant flowers known for their characteristic aromas. A total of 65 volatile compounds were identified in jujube flowers at different stages. The aroma fingerprint analysis revealed 24 distinct aromas, with 14 aromas increasing in intensity from the bud stage to full bloom. Varieties such as Fuxiang, Dongzao, and Xingguang exhibited significantly stronger aromas at the flowering stage compared to other varieties. Additionally, sensory evaluation indicated a preference for the fragrance of jujube flowers among men. This study offers new insights into the development of jujube flower-based spices, highlighting their considerable potential and laying a foundation for further research on plant flower spices.

## Highlights

- The volatile fingerprints of jujube flowers in different periods were established by HS-GC-IMS.
- HS-GC-IMS combined with PCA can well differentiate the volatile differences of jujube flowers in different periods.
- Sensory evaluation showed men prefer preferred jujube aroma.
- Jujube flowers aroma has the potential to be developed into men ‘s perfume.

## 1 Introduction

Jujube (*Ziziphus jujuba* Mill.) belongs to the genus *Ziziphus* of the family Rhamnaceae (Gao et al., 2013). It is native to China and is cultivated in Asia, Europe and the Americas (Liu et al., 2020). China has the largest the world’s largest jujube cultivation area globally, with a history over 4,000 years (Hernández et al., 2015; Li et al., 2009; Wojdyło et al., 2016). Jujube fruit contains vitamins, carbohydrates, organic acids, cAMP, and proline, making it both a medicinal and edible treasure (Choi et al., 2011; Guo et al., 2015; Li et al., 2007; Liu et al., 2015). The jujube flower is the primary source of fragrance in the jujube tree, and it is highly esteemed for its pleasant aroma and abundance. It also provides valuable nectar, making it important to investigate the fragrance attributes of jujube flowers.

Generally, Jujube flowers bloom in May and can last until July. Jujube flowers are small and numerous, exhibiting a yellow-green color as a whole, with a uniquely warm and inviting fragrance.

Flavor is an important perceptual property of foods, and it that is used in assessing their nutrition-related properties and freshness (Shi et al., 2017). Floral scents are primarily derived from VOCs synthesized within plant organs and released into the air. Generally, Jujube flowers bloom in May and can last until July. Jujube flowers are small and numerous, exhibiting a yellow-green color as a whole, with a uniquely warm and inviting fragrance. The aroma of jujube flowers serves as a signal to attract honey bees, which collect nectar to produce jujube honey after its own transformation process (Ma W. et al., 2020). Jujube flowers contain many volatile compounds, including esters, alkanes, aldehydes, and ketones, which contribute to their pleasant aroma (Xia et al., 2020). Furthermore, these flowers contain essential nutritional components, including calcium (Ca), iron (Fe), magnesium (Mg), as well as bioactive compounds such as mannose and phenols, making them a unique blend of scent and nutrition (Du et al., 2024; Fahim et al., 2014).

Volatile compounds are crucial not only for the fragrance of plants but also for their ecological interactions and potential applications in flavor, fragrance, and health-related industries. As the cultivation area of jujube continues to expand, understanding the aromatic characteristics of jujube flowers becomes increasingly important. The fragrance of jujube flowers, known for its delicate, restrained, and enduring profile, has long been appreciated, particularly in East Asian cultures, where it aligns with traditional preferences. This subtle yet distinctive aroma holds promising potential for both commercial and cultural applications. While previous studies have primarily focused on fruit aroma (Feng et al., 2022), the volatile compounds in flowers, and their dynamic changes throughout development and in different varieties, have not been systematically explored. Moreover, there is a significant gap in research comparing the volatile components of jujube within other flowers.

The study of plant aroma compounds plays a crucial role in understanding plant sensory characteristics, with aroma being a key distinguishing feature of jujube. Jujube aroma consists of a complex mixture of volatile compounds produced through various biotransformations of sugars, amino acids and other metabolites (Wang et al., 2018; Zhu and Xiao, 2018). Due to its ease of operation, enhanced sensitivity, streamlined processing, and efficient analytical speed, gas chromatography-ion mobility spectometry (GC-IMS) has become a widely used method food aroma analysis (Wang et al., 2020). Different plant tissue exhibit unique flavor profile, with flowers often having the most pronounced aromas (Ma et al., 2023). GC-IMS is a novel technique for odors detection, with IMS separating ions based on their mobility under atmospheric pressure (Li et al., 2019). Combining IMS with other analytical instruments enhances its performance and improves the accuracy of results. In recent years, headspace gas chromatography-ion mobility spectrometry (HS-GC-IMS) has been widely applied to investigate volatile compounds in food science. For example (Feng et al., 2022), used GC-IMS and sensory evaluation to characterize the volatile organic compounds (VOCs) characteristics in Molixiang grapes from different regions. In another study (Leng et al., 2021), employed GC-IMS to analyze postharvest preservation of peach fruit, finding a significant increase in esters and alcohols levels with prolonged storage. Similarly (Yao et al., 2024), examined the volatile components and aroma profiles of Yijiangzi monofloral honey and its corresponding flowers by GC-IMS, discovering that most of the compounds in honey originated from flowers. PCA is a detection method based on multivariate statistics, which is used to reduce the dimension or transform multiple indicators into several comprehensive indicators for extracting features and revealing the relationship between variables. The converted score information also can be entered into a cluster analysis and further discriminant analysis. PCA can be combined with HS-GC-IMS to detect the volatile flavor substances in plant flower. Based on these previous studies, GC-IMS appears to be a promising technique for analyzing VOCs in jujube flowers.

This study aims to fill these knowledge gaps by systematically investigating the volatile components of jujube flowers. Flowers in three development stages and eleven varieties were studied to reveal the distribution of volatile compounds and identify the characteristic components. Furthermore, jujube flowers were compared with other plant species renowned for their distinctive floral aromas, providing a comprehensive orientation towards understanding the diversity of floral fragrance profiles. The findings from this research will provide valuable insights for the development and application of jujube flower aroma and serve as a reference for future studies on the volatile compound accumulation mechanisms in jujube. This work will contribute to the broader understanding of plant volatiles and their potential in various industries.

## 2 Materials and methods

### 2.1 Plant material

**Figure 1:**
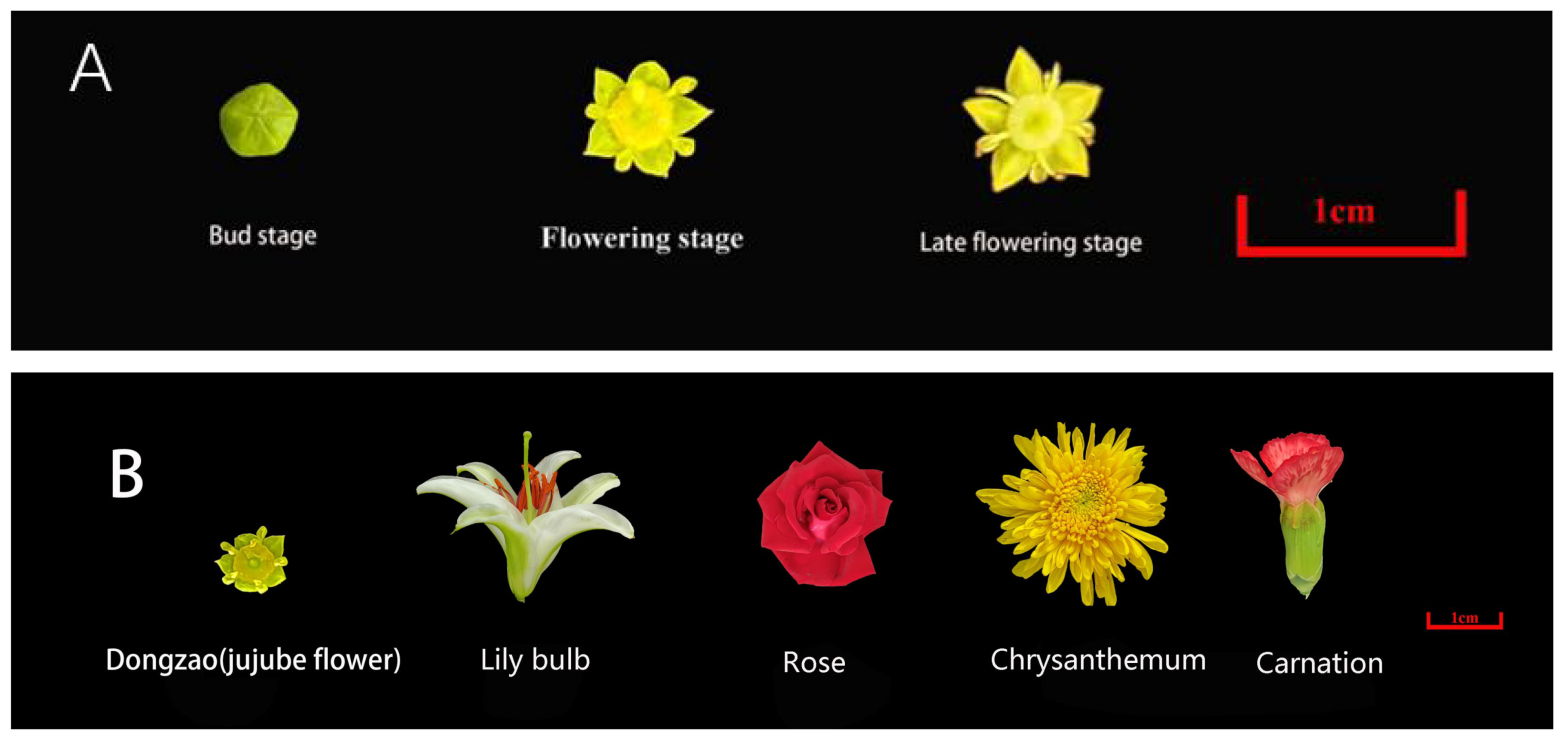
**(A)** Jujube flowers in three periods. **(B)** Jujube flowers and four other commonly recognized flowers.

All jujube flowers samples used in this study were collected from the Third Farm Branch of Hebei Agricultural University (115°48′E, 38°85′N) in June 2023. Samples were harvested at three developmental stages: flower bud stage, flowering stage, and late flowering stage, and immediately transported to the laboratory for analysis. Jujube flowers with uniform appearance and no physical damage were selected as experimental samples (**Figure 1**). The study included eleven jujube varieties: Fushuai, Fuxiang, Xingguang, Dongzao, Yueguang, Yuhong, Chenguang, Yushuai, Zanshuo, Jinsixiaozao, and Dajinsiwang. For comparative analysis, flowers of Lily bulb, Rose, Chrysanthemum, and Carnation were obtained from the Baoding Agro-ecological Park in Hebei Province (**Figure 1**). These four species were selected due to their widespread recognition and distinctive aromatic properties. All samples were healthy, free from pest damage, and exhibited no signs of spoilage.

### 2.2 Sensory evaluation

The sensory evaluation was conducted in accordance with the ethical guidelines outlined in the World Medical Association (Helsinki Declaration). A total of 20 healthy, non-smoking participants (10 men and 10 women, aged 20–42 years) were recruited from students and faculty members of our institution. Prior to the aroma evaluation, all participants underwent a one-week training program, which included more than two hours of daily sessions to familiarize them with the characteristics of plant floral aromas and the requirements for sensory evaluation. During this training, participants were introduced to the descriptive terminology used for plant floral aromas. Written informed consent was obtained from each participant before commencing the sensory evaluation.

### 2.3 Headspace gas chromatography-ion mobility spectrometry analysis

The volatile compounds of jujube flowers were analyzed using HS-GC-IMS flavor analyzer (Flavour Spec ^®^) provided by the company für Analytische Sensorysteme mb H (G.A.S., Dortmund, Germany). For the analysis, 1g of flower sample was weighed and placed in a 20ml headspace glass extraction bottle. The samples were incubated at 60°C for 15 min. Following incubation, 0.5 ml of the headspace phase was automatically injected into the sampler in a splitless injection mode with a syringe at 45°C. Volatile components were separated using an MXT-WAX capillary column (30 m × 0.53 mm ID, 1.0 μm df, RESTEK, USA) in the gas chromatography and coupled to IMS at 45°C. Nitrogen (purity of 99.999%) was used as the carrier gas. The program flow rate was as follows: 2 mL/min for the first 2 min, increasing linearly to 10 mL/min over 8 min, then increasing to 100 mL/min over the final 15 minutes, after which the flow was stopped. The analysis was conducted under normal pressure. The analyte was separated on a column maintained at 60°C and ionized in the IMS ionization chamber at 45°C. The drift gas (nitrogen) flow rate was set to 150 mL/min. Each sample was analyzed in triplicate. The retention index (RI) of volatile compounds was calculated using C4-C8 n-ketone (China National Pharmaceutical Chemical Reagents Beijing Co., Ltd.) as an external reference under the same chromatographic conditions. Volatile compounds were identified by comparing the standard drift time and RI with those in the GC-IMS libraries and NIST 2014 (National Institute of Standards and Technology, Gaithersburg, Maryland, United States).

### 2.4 Statistical analysis

The HS-GC-IMS data is processed using the laboratory analysis instrument Vocal and its three plug-ins, the NIST database and the IMS database. The topographic map and fingerprint of volatile compounds in jujube flowers were established by using the Reporter and Gallery Plot plug-in. Principal Component Analysis (PCA) was performed using the Dynamic PCA plug-in (g.a.s., Dortmund, Germany) to evaluate the regularity and difference between test samples. The analysis was performed using Origin software (version 2021b, USA). OPLS-DA and PLSR analyses were conducted to further evaluate the characteristics of samples using SIMCA 14.1.

## 3 Results and discussion

### 3.1 Variation of volatile flavor compounds in jujube flowers at different growth stages

#### 3.1.1 HS-GC-IMS topographic maps of jujube flowers across different growth stage

**Figure 2:**
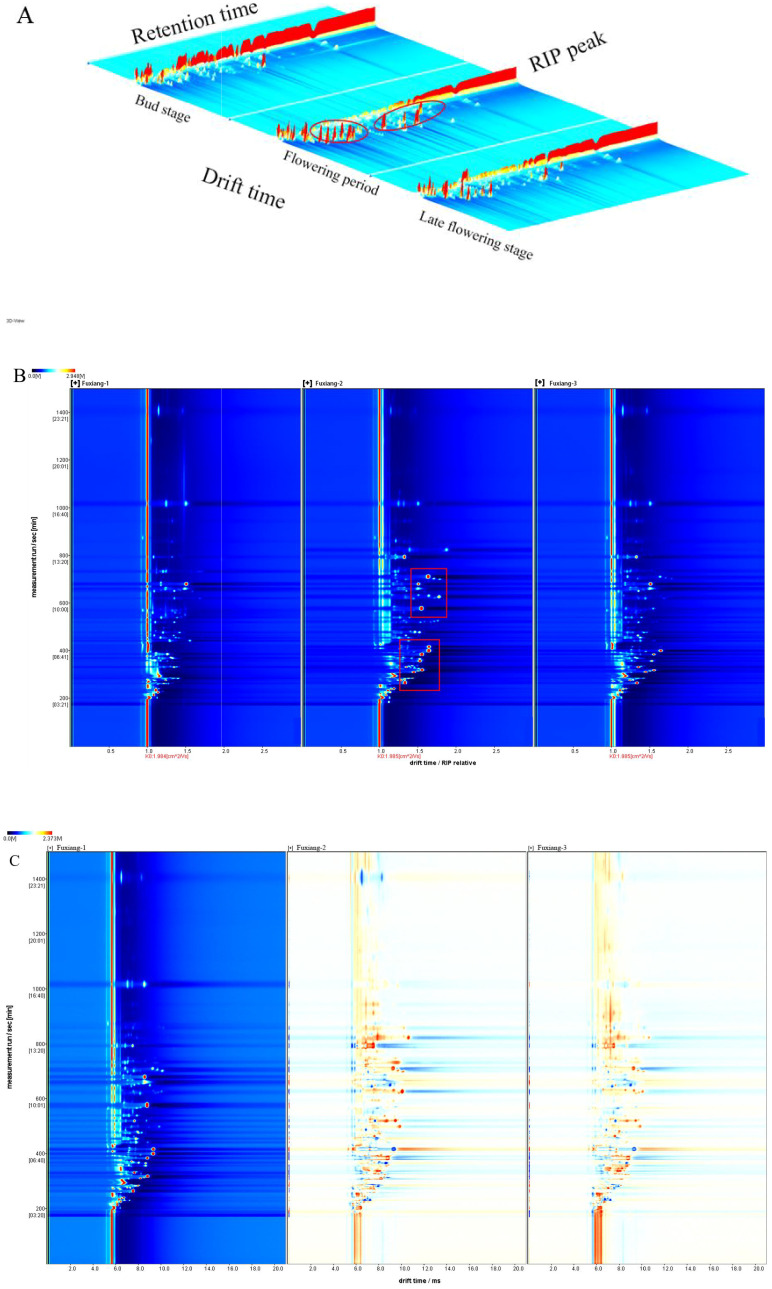
GC-IMS analysis of Fuxiang flowers in different periods. **(A)** 3D-topographic plots of Fuxiang flowers; **(B)** 2D-topographic plots of Fuxiang flowers; **(C)** The difference comparison topographic plots of Fuxiang flowers.

HS-GC-IMS was employed to analyze the differences in volatile substances in jujube flowers at various growth stages. Taking the Fuxiang variety as an example, **Figure 2** showed the topographic map of volatile substances, where X, Y and Z represent ion migration time, GC retention time and ion peak intensity, respectively. As illustrated in **Figure 2**, jujube flowers at different stages showed similar visualization patterns, with slight variations in ion peak intensity. The red-circled area in **Figure 2** clearly highlights the differences in peak intensity between the various stages. Notably, the ion peak intensity during the flowering stage was significantly higher compared to the bud stage and the late flowering stage. GC-IMS separates mixtures into individual compounds through a gas chromatography column, then ionizes these compounds using ion mobility spectrometry, and further analyzes them based on the migration rates of each compound’s ions under the influence of an electric field (Yang et al., 2021). This dual separation allows for the distinct identification of each compound. By analyzing the migration time and ion peak intensity of each volatile compound, the volatile profile of jujube flowers can be qualitatively analyzed.

Because the topographic map is relatively rough, the volatile substances in jujube flowers cannot be clearly displayed. To better distinguish the volatile flavor components at different stages of flowering, we normalized the ion migration time and reaction ion peak (RIP). Each point around the RIP represents a volatile substance found in jujube flowers. Most volatile substance signals appeared within a drift time of 1.0-1.75 ms and a retention time of 200-1000 s. In the visualization, the color of each point indicates the signal intensity of a single substance: red indicates a higher intensity, blue signifies lower intensity, and the color depth correlated with the intensity level. Different models were applied to distinguish the volatile components of jujube flowers across various growth stages, as shown in **Figure 2**. From the two-dimensional topographic plots (**Figure 2**), it is evident that the signal intensities within the red box are markedly higher in jujube flowers during the flowering stage compared to those at the other two developmental periods. In **Figure 2**, the bud stage of Fuxiang variety is used as a reference to compare the other two stages. Red indicates a higher intensity than the reference, while blue indicates a lower intensity. When the drift time was 1.0-1.5 ms and the retention time was 400-800 s, the volatile substances in both the flowering stage and the late flowering stage were significantly higher than those in the bud stage, with the flowering stage showing slightly more red than that in the late flowering stage. As jujube flowers continue to bloom, certain aroma components are continuously produced. However, during the late flowering stage, the levels of these aroma components gradually decline as a result of flower ovary pollination and the subsequent formation of fruits. To further explore the volatile substances represented by these red dots, an in-depth statistical analysis of jujube samples across different periods is necessary.

#### 3.1.2 Qualitative analysis volatile flavor components in jujube flowers at different growth stages

**Figure 3:**
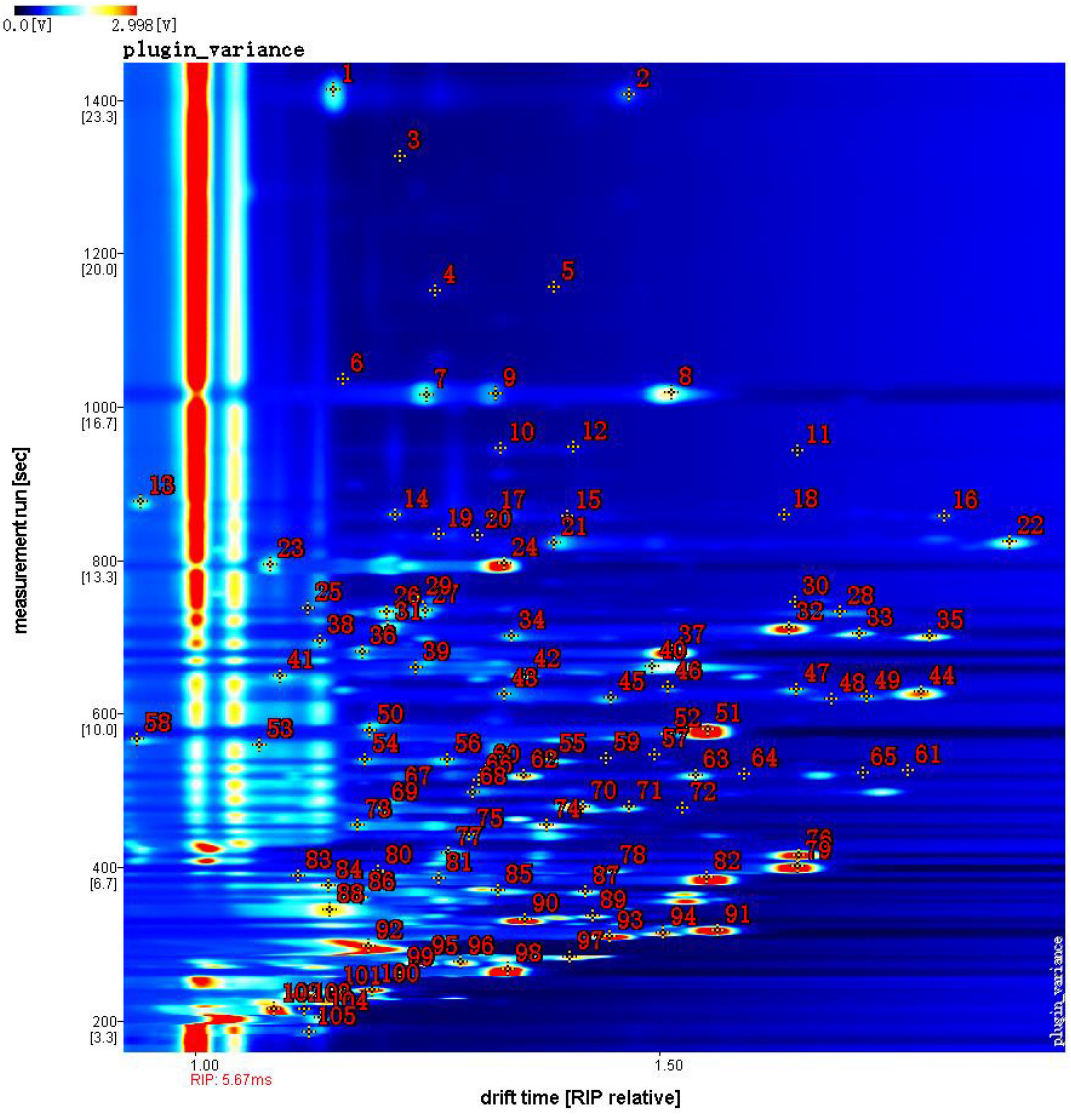
Library search qualitative analysis of jujube flowers across different periods.

In this study, the two-dimensional cross-qualitative approach, utilizing the GC-IMS Library Search, was employed to further identify the detected compounds. **Figure 3** presents the results of the library search-based qualitative analysis of the sample. In **Figure 3**, the horizontal axis represents the drift time, while the vertical axis corresponds to the retention time. The red numbers displayed in **Figure 3**, denote the identified organic compounds. **Table 1** provides the qualitative results of volatile components detected in jujube flowers across different growth stages. The red numbers in **Figure 3**, align with the compound numbers listed in **Table 1**. Notably, due to the enhanced proton affinity and higher concentration of dimers, their drift time is greater than that of monomers (Lantsuzskaya et al., 2015). Some compounds may produce multiple signals due to varying concentrations, such as monomers and dimers (Li et al., 2019). According to our study results, we found that a total of 31 compounds exist in the form of dimers. Ultimately, we detected 105 peaks and identified 65 volatile compounds from our samples, including 15 aldehydes, 12 esters, 11 alcohols, 11 olefins, 8 ketones, 4 ethers, and 4 other substances (1 furan, 1 pyrazine, 1 thiazole and 1 pyridine), as show in **Table 1**.

**Table 1:** Detected volatile organic compounds (VOCs) in jujube flowers identified byHS-GC-IMS.

Count	Compound	Flavor description	CAS#	Formula	MW^a^	RI^b^	Rt^c^	Dt^d^	Comment
Aldehydes									
1	Benzaldehyde	sweet, almond, nutty	C100527	C7H6O	106.1	1493	1413.836	1.15015	Monomer
2	Benzaldehyde		C100527	C7H6O	106.1	1491.6	1408.328	1.46858	Dimer
3	n-Octanal	fatty, citrus, honey	C124130	C8H16O	128.2	1316.1	858.444	1.40149	Monomer
4	n-Octanal		C124130	C8H16O	128.2	1315.8	857.796	1.80804	Dimer
5	(E)-2-Heptenal	guava	C18829555	C7H12O	112.2	1266.6	746.689	1.24164	Monomer
6	(E)-2-Heptenal		C18829555	C7H12O	112.2	1266.1	745.634	1.64713	Dimer
7	Butanal	fermented, bready	C123728	C4H8O	72.1	827.3	216.273	1.08541	Monomer
8	Butanal		C123728	C4H8O	72.1	825.2	215.009	1.11821	Dimer
9	Valeraldehyde	cocoa chocolate	C110623	C5H10O	86.1	1008	360.018	1.17985	Monomer
10	Valeraldehyde		C110623	C5H10O	86.1	1017.1	369.369	1.42174	Dimer
11	1-hexanal	green, woody, vegetative	C66251	C6H12O	100.2	1032.8	386.144	1.26358	Monomer
12	1-hexanal		C66251	C6H12O	100.2	1033.6	386.969	1.552	Dimer
13	3-Methyl-2-butenal	nutty, cherry	C107868	C5H8O	84.1	1217.3	649.704	1.09243	Monomer
14	3-Methyl-2-butenal		C107868	C5H8O	84.1	1217.8	650.577	1.35719	Dimer
15	2-Hexenal	almond, apple, plum	C505577	C6H10O	98.1	1234.1	681.148	1.18116	Monomer
16	2-Hexenal		C505577	C6H10O	98.1	1235	682.895	1.51462	Dimer
17	(E)-2-hexen-1-al	green, apple	C6728263	C6H10O	98.1	1209.6	635.728	1.51032	
18	Butyraldehyde	bready, yeasty	C123728	C4H8O	72.1	915.5	277.32	1.28767	
19	2-methylbutanal	green, fruity	C96173	C5H10O	86.1	922.3	282.722	1.40501	
20	3-Methyl butanal	peach	C590863	C5H10O	86.1	864.8	240.412	1.19159	
21	Octylaldehyde	orange	C124130	C8H16O	128.2	1351.3	948.006	1.40896	
22	Propanal	nutty	C123386	C3H6O	58.1	861.2	237.999	1.15131	
23	(E)-2-Pentenal	sharp	C1576870	C5H8O	84.1	1165.2	560.897	1.36286	
Alcohols									
24	3-heptanol	green, herbal	C589822	C7H16O	116.2	1350.9	946.885	1.32951	Monomer
25	3-heptanol		C589822	C7H16O	116.2	1349.6	943.52	1.64988	Dimer
26	3-Methylbutan-1-ol	green, fruity	C123513	C5H12O	88.1	1223.5	661.059	1.23841	Monomer
27	3-Methylbutan-1-ol		C123513	C5H12O	88.1	1224.2	662.369	1.49315	Dimer
28	1 -hexanol	peach, fruity, green, sweet	C111273	C6H14O	102.2	1316.2	858.734	1.32117	Monomer
29	1 -hexanol		C111273	C6H14O	102.2	1316.4	859.094	1.63589	Dimer
30	Butanol	fruity, green, sweet	C71363	C4H10O	74.1	1152.3	540.792	1.18445	Monomer
31	Butanol		C71363	C4H10O	74.1	1152.3	540.792	1.38521	Dimer
32	1-Pentanol	wintergreen, almond	C71410	C5H12O	88.1	1151.9	540.215	1.27273	Monomer
33	1-Pentanol		C71410	C5H12O	88.1	1156.4	547.132	1.49627	Dimer
34	3-Pentanol	sweet, nutty	C584021	C5H12O	88.1	1107.9	477.171	1.20437	Monomer
35	3-Pentanol		C584021	C5H12O	88.1	1108.9	478.546	1.41842	Dimer
36	1- butanol	butter, creamy	C71363	C4H10O	74.1	1091.6	455.72	1.17545	Monomer
37	1- butanol		C71363	C4H10O	74.1	1092	456.27	1.37988	Dimer
38	2-butanol	fruit, green	C78922	C4H10O	74.1	1024.6	377.344	1.14496	Monomer
39	2-butanol		C78922	C4H10O	74.1	1017.6	369.919	1.32754	Dimer
40	Propanol	camphor	C71238	C3H8O	60.1	1035.6	389.169	1.11239	
41	Linalool	citrus, rose, woody, green	C78706	C10H18O	154.3	1470.7	1327.537	1.22144	
42	1 -heptanol	onion, sweet, corn, green	C111706	C7H16O	116.2	1421.8	1156.775	1.38779	
Alkenes									
43	(E)-3-hexen-1-ol	green, cherry	C928972	C6H12O	100.2	1376	1016.412	1.25006	Monomer
44	(E)-3-hexen-1-ol		C928972	C6H12O	100.2	1376.8	1018.655	1.51404	Dimer
45	beta-ocimene	green, woody	C13877913	C10H16	136.2	1260.4	733.663	1.20741	Monomer
46	beta-ocimene		C13877913	C10H16	136.2	1260.7	734.33	1.24923	Dimer
47	beta-ocimene		C13877913	C10H16	136.2	1260.1	732.996	1.69536	Trimer
48	Limonene	lemon, fruity	C138863	C10H16	136.2	1249.9	712.156	1.20836	Monomer
49	Limonene		C138863	C10H16	136.2	1250.3	713.03	1.64056	Dimer
50	Limonene		C138863	C10H16	136.2	1245.9	704.295	1.71641	
51	3-carene	orange, fruity	C13466789	C10H16	136.2	1207.6	632.234	1.64914	Monomer
52	3-carene		C13466789	C10H16	136.2	1200.5	619.569	1.68635	Dimer
53	3-carene		C13466789	C10H16	136.2	1202.1	622.512	1.72438	Trimer
54	myrcene	peach, fruity, green	C123353	C10H16	136.2	1139.9	522.271	1.59313	Monomer
55	myrcene		C123353	C10H16	136.2	1140.8	523.646	1.7204	Dimer
56	(Z)-2-pentenol	fruity, green	C1576950	C5H10O	86.1	1323.4	876.236	0.94252	
57	alpha-terpinolene	sweet, floral, plastic	C586629	C10H16	136.2	1316.6	859.74	1.21715	
58	2-methyl-2-propenal	fruity, green, sweet	C78853	C4H6O	70.1	897.3	263.507	1.22167	
59	1-Penten-3-ol	fruity, green	C616251	C5H10O	86.1	1169.2	567.306	0.93813	
60	beta-Pinene	woody, camphor	C127913	C10H16	136.2	1082	443.62	1.29614	
61	1,4-dimethylbenzene	pungent, fruity	C106423	C8H10	106.2	1164.5	559.813	1.07054	
Ester									
62	Hexanoic acid propyl ester	fruity	C626777	C9H18O2	158.2	1301.5	823.858	1.38732	Monomer
63	Hexanoic acid propyl ester		C626777	C9H18O2	158.2	1301.7	824.218	1.87885	Dimer
64	amyl acetate	banana, pear	C628637	C7H14O2	130.2	1142.7	526.396	1.31429	Monomer
65	amyl acetate		C628637	C7H14O2	130.2	1143.1	526.946	1.769	Dimer
66	Ethyl 2-methy lpropionate	sweet, floral	C97621	C6H12O2	116.2	940.8	297.847	1.18835	Monomer
67	Ethyl 2-methy lpropionate		C97621	C6H12O2	116.2	964.3	318.281	1.5639	Dimer
68	isoamyl formate	wine, fruity	C110452	C6H12O2	116.2	1061.7	418.87	1.27405	Monomer
69	Isoamyl formate		C110452	C6H12O2	116.2	1059.3	416.12	1.65201	Dimer
70	ethyl (E)-2-butenoate	spicy, sweet, garlicy	C623701	C6H10O2	114.1	1176	578.258	1.18872	Monomer
71	ethyl (E)-2-butenoate		C623701	C6H10O2	114.1	1177.4	580.564	1.55322	Dimer
72	Methyl butyrate	fruity, green	C623427	C5H10O2	102.1	993.4	345.526	1.14632	Monomer
73	Methyl butyrate		C623427	C5H10O2	102.1	985	337.405	1.42968	Dimer
74	2-Methyl butanoic acid ethyl ester	apple, fruity	C7452791	C7H14O2	130.2	1046.7	401.544	1.64969	
75	isovaleric acid, methyl ester	fruity, celery	C556241	C6H12O2	116.2	1042	396.319	1.19845	
76	(E)-Ethyl-2-hexenoate	fruity, vegetable	C27829727	C8H14O2	142.2	1376.4	1017.533	1.32439	
77	Ethyl propanoate	sweet, rum, fruit, grape, pineapple	C105373	C5H10O2	102.1	956	310.946	1.4473	
78	3-methylbutyl propanoate	fruity, green	C105680	C8H16O2	144.2	1138.8	520.621	1.35478	
79	Acetic acid ethyl ester	fruity, nutty	C141786	C4H8O2	88.1	902.4	267.314	1.33738	
Ketone									
80	2,3-Pentanedione	spicy, butter, creamy	C600146	C5H8O2	100.1	1122.9	497.796	1.21941	Monomer
81	2,3-Pentanedione		C600146	C5H8O2	100.1	1123.5	498.621	1.3004	Dimer
82	Isovalerone	green, fruity, sweet	C108838	C9H18O	142.2	1244.4	701.238	1.34145	Monomer
83	Isovalerone		C108838	C9H18O	142.2	1244.8	702.112	1.79226	Dimer
84	2,6-dimethyl-4-heptanone	oily, woody, lemon, herbal	C108838	C9H18O	142.2	1204.4	626.557	1.33429	Monomer
85	2,6-dimethyl-4-heptanone		C108838	C9H18O	142.2	1205.7	628.74	1.78367	Dimer
86	6-methyl-5-hepten-2-one	citrus, fruity	C110930	C8H14O	126.2	1383	1036.597	1.16036	
87	2-Butanone	green, clover, honey, sweet	C78933	C4H8O	72.1	914.2	276.366	1.24801	
88	Hexan-2-one	fruity, green, nutty	C591786	C6H12O	100.2	1173.5	574.223	1.50908	
89	3-penten-2-one, 4-methyl	Mint, honey	C141797	C6H10O	98.1	1153.4	542.548	1.44335	
90	2-propanone	oily, woody, lemon, herbal	C67641	C3H6O	58.1	772.6	185.353	1.12322	
Others									
91	2-Ethoxyethanol	sweet, minty	C110805	C4H10O2	90.1	1288.7	794.555	1.08235	Monomer
92	2-Ethoxyethanol		C110805	C4H10O2	90.1	1289.3	795.993	1.33379	Dimer
93	diethyl disulfide	onion	C110816	C4H10S2	122.2	1241.3	695.124	1.13537	
94	Dimethyl trisulfide	minty, herbal, eucalyptus	C3658808	C2H6S3	126.3	1305.2	832.487	1.30528	
95	Dipropyl disulfide		C629196	C6H14S2	150.3	1420.2	1151.266	1.25947	
96	2-ethylpyrazine	peanuts, nuts, roasted cocoa, woody	C13925003	C6H8N2	108.1	1262.6	738.332	1.12221	
97	2,6-Lutidine	nuts, amines, wood, bread	C108485	C7H9N	107.2	1201.2	620.879	1.44879	
98	tetrahydrothiophene	pungent, fruity	C110010	C4H8S	88.2	1134.1	513.746	1.30619	
99	2,5-Dimethylfuran	green, sweet	C625865	C6H8O	96.1	978.9	331.642	1.35647	
Unknowns									
100	1		unidentified	*	*	1138.6	520.346	1.53991	
101	2		unidentified	*	*	1109.5	479.371	1.46817	
102	3		unidentified	*	*	1108.5	477.996	1.52602	
103	4		unidentified	*	*	1041.8	396.044	1.45082	
104	5		unidentified	*	*	960.2	314.614	1.5056	
105	6		unidentified	*	*	806.3	203.859	1.13657	

a Represents the molecular mass.

b Represents the retention index calculated using n-ketones C4–C8 as external standard on MXT-WAX column.

c Represents the retention time in the capillary GC column.

d Represents the drift time in the drift tube.

#### 3.1.3 Analysis of volatile flavor components of jujube flowers in different periods

**Figure 4:**
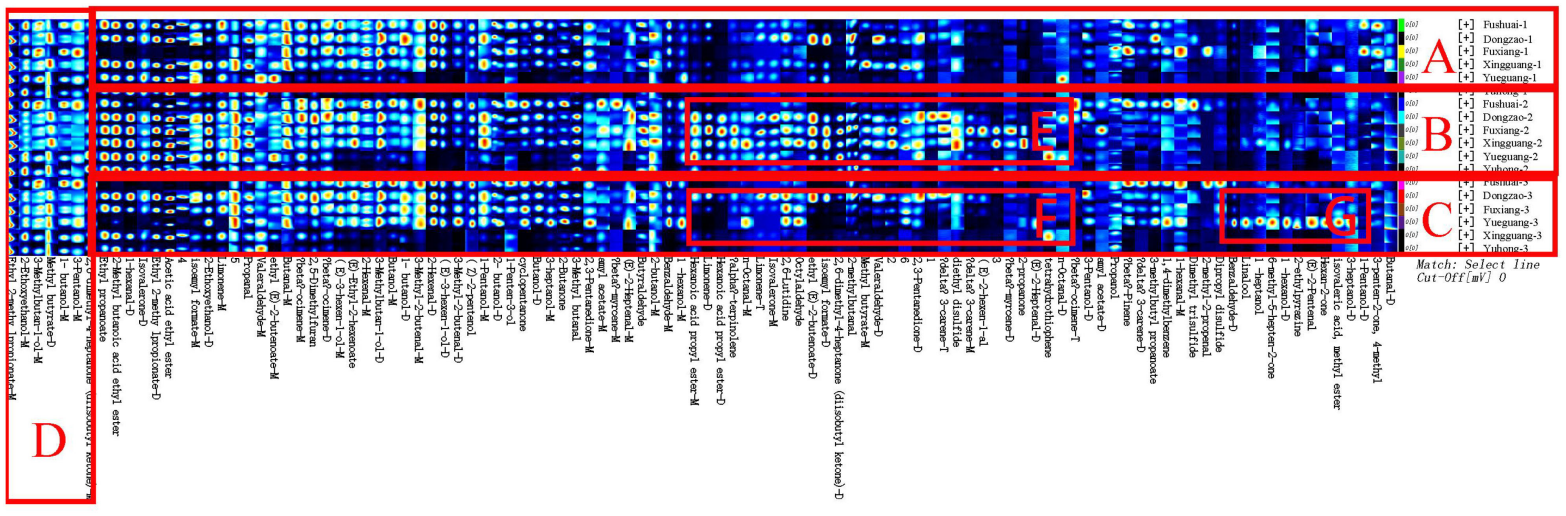
Fingerprint comparison of volatile organic compounds (VOCs) in jujube flowers samples determined by HS-GC-IMS.

To distinguish the differences between jujube flowers in different periods, the Gallery Plot plug-in was used to generate volatile fingerprints (**Figure 4**). The row represents the detected volatile organic compounds, and the column showed the signal intensity of the same compound presented in different jujube flowers samples. Each point in the figure represents a volatile substance, and the depth of the color is related to the content of the volatile substance. The brighter the color, the higher the content of the substance in the sample.

As illustrated in **Figure 4**, the aromatic profiles of jujube flowers were compared across different growth stages. The highest diversity of aroma components was observed during the flowering stage (**Figure 4**, red box B), while the bud stage exhibited the least variety (**Figure 4**, red box A). Common to all samples were compounds such as methyl butyrate, 1-butanol, 3-pentanol, 2,6-dimethyl-4-heptanone, ethyl 2-methylpropionate, 2-ethoxyethanol, and 3-methylbutan-1-ol (**Figure 4**, red box D). During the bud stage, the aroma profile was predominantly characterized by a higher presence of alcohols and aldehydes (**Figure 4**, red box A). Notably, compounds like 3-heptanol, 3-methylbutan-1-ol, n-octanal, benzaldehyde, 2-butanol, butyraldehyde, 2-methylbutanal, and 3-methylbutanal were found in higher concentrations, imparting grassy, nutty, and jasmine-like scents to the jujube flowers. As the flower buds developed and accumulated nutrients, the aromatic intensity of the flowers gradually increased.

The flowering stage marked a significant divergence in aroma composition compared to the other stages, with an increase in 14 distinct aroma substances, including valeraldehyde, (E)-2-hexen-1-al, octylaldehyde, limonene, 3-carene, myrcene, terpinolene, hexanoic acid propyl ester, isoamyl formate, ethyl (E)-2-butenoate, 2,3-pentanedione, 2,6-dimethyl-4-heptanone, 2-propanone, and diethyl disulfide (**Figure 4**, red box E). These newly emerged compounds were primarily esters and alkenes. Substances such as myrcene, terpinolene, 2-nonanone, 2,3-pentadione, and phellandrene, which are yellow liquids under standard conditions, contribute to the yellow coloration of the nectar disc and enhance the flowers’ robust fragrance.

During the late flowering stage, some of the aroma components that increased during the flowering stage may have oxidized due to prolonged exposure to air. Consequently, the newly formed aroma components from the flowering period were largely absent in the late flowering stage (**Figure 4**, red box F). However, as indicated in **Figure 4**, red box G, eight additional aroma components were identified in the late flowering stage, including dipropyl disulfide, 2-methylbutanal, linalool, 6-methyl-5-hepten-2-one, 1-heptanol, (E)-2-pentenal, isovaleric acid, and methyl ester. Similar compounds, such as Benzaldehyde, (E) -2-Heptenal, and Butanal, have also been found in jujube fruit (Yang et al., 2019).

**Figure 5:**
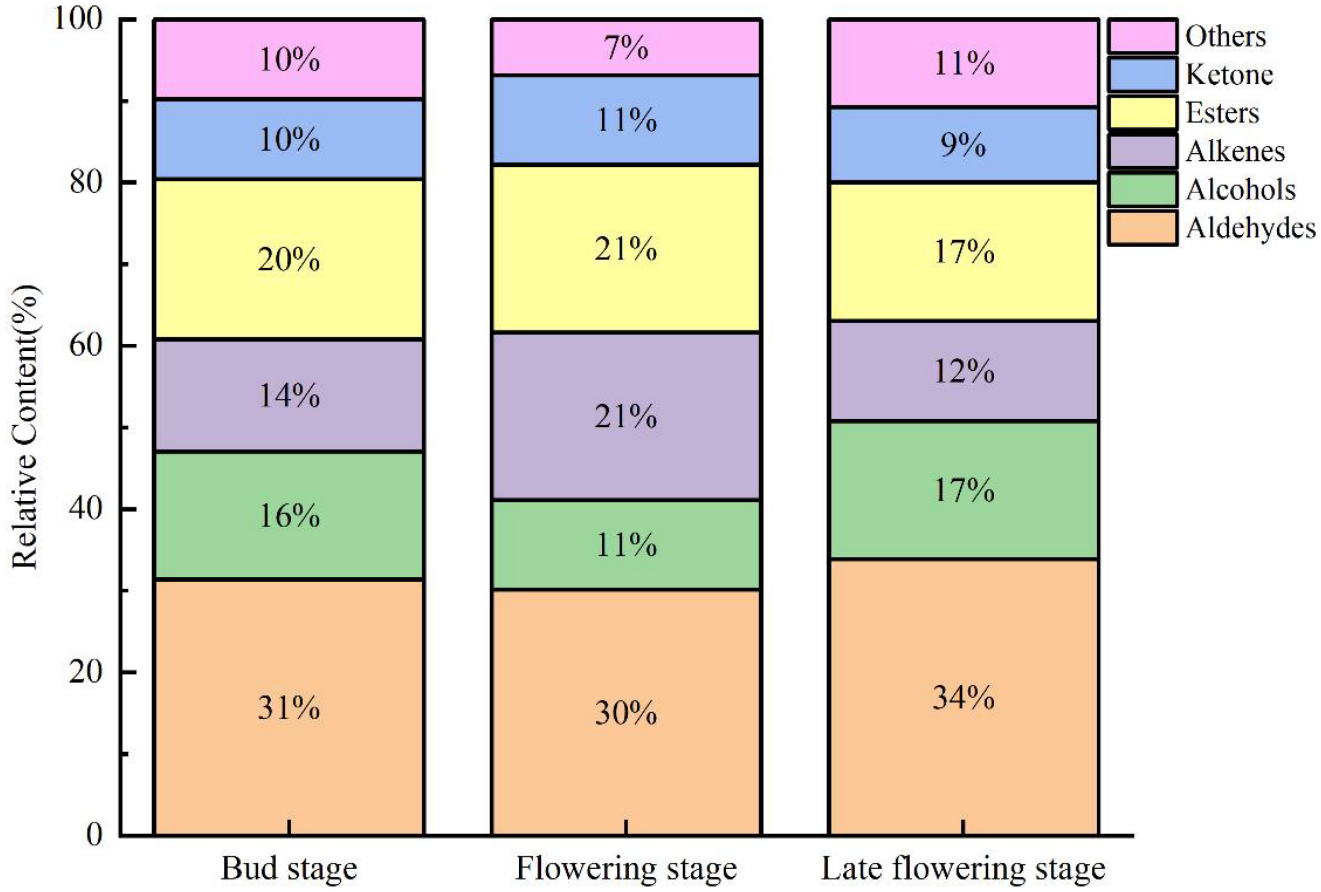
Relative contents of volatile components in different periods of jujube flowers.

Similarly, aldehydes were found to be the most abundant across all three stages, followed by esters, alkenes, alcohols, and ketones (**Figure 5**). Notably, the levels of esters and alkenes during the flowering stage were significantly higher compared to the other two stages, serving as the primary factor contributing to the differences observed across the periods. In contrast, ketones and aldehydes exhibited minimal changes during the flowering stage, suggesting that they have a limited influence on the aroma profile of jujube flowers. During the flowering bud stage, a higher concentration of aldehydes and alcohols was formed, which were subsequently transformed into esters and alkenes during the flowering stage, thereby enhancing the aromatic profile of jujube flowers. The disappearance of certain compounds in the late flowering stage may be attributed to factors such as bee pollination, flower ovary fertilization, and oxidation due to prolonged exposure to air. Comparable findings have been reported in studies on truffles, red peppers, green peppers, and tea (Korkmaz et al., 2023; Shao et al., 2023; Yue et al., 2023).

#### 3.1.4 PCA of volatile flavor components in jujube flowers across different developmental stages

**Figure 6:**
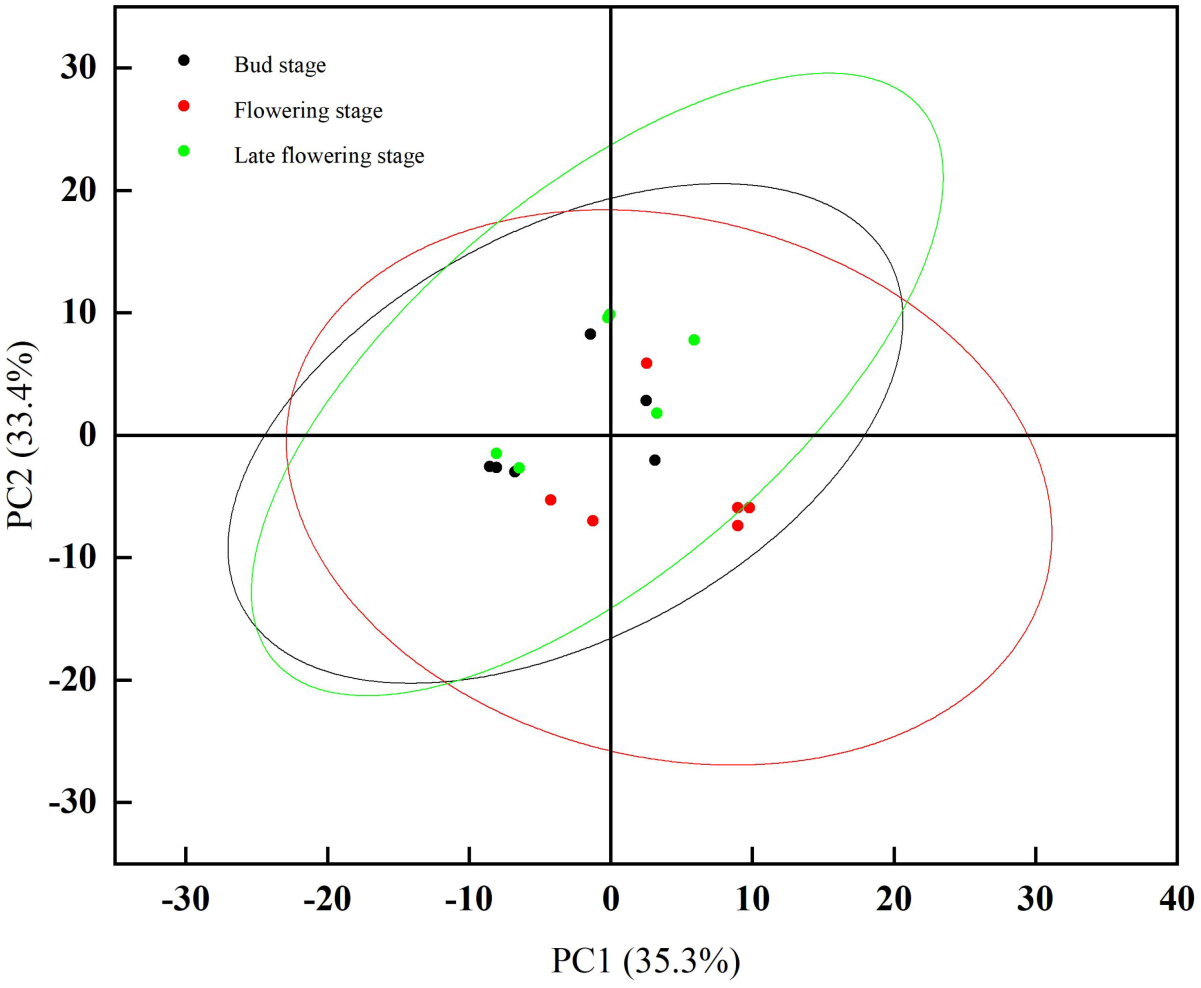
PCA analysis results of volatile components of jujube flowers in different periods.

Principal Component Analysis (PCA) is a widely utilized unsupervised analytical tool for the analysis of volatile compounds. In a PCA plot, samples with similar expression profiles exhibit a higher degree of clustering, while those with greater differences are positioned farther apart (Ma Q. et al., 2020). To better distinguish and comprehensively analyze the variations in volatile compounds across different stages of jujube flowers, we applied principal component analysis. As shown in **Figure 6**, the combined contribution of PC1 and PC2 for jujube flowers across different stages exceeds 60%, which is sufficient to highlight the distinctions among the stages. The aroma profile of jujube flowers at the flowering stage is notably distant from those at the bud stage and the late flowering stage, indicating a significant difference between the flowering stage and the other two periods. This finding is consistent with the results derived from the fingerprint analysis, as previously discussed.

### 3.2 Volatile flavor components variations among different jujube flowers varieties at the flowering stage

**Figure 7:**
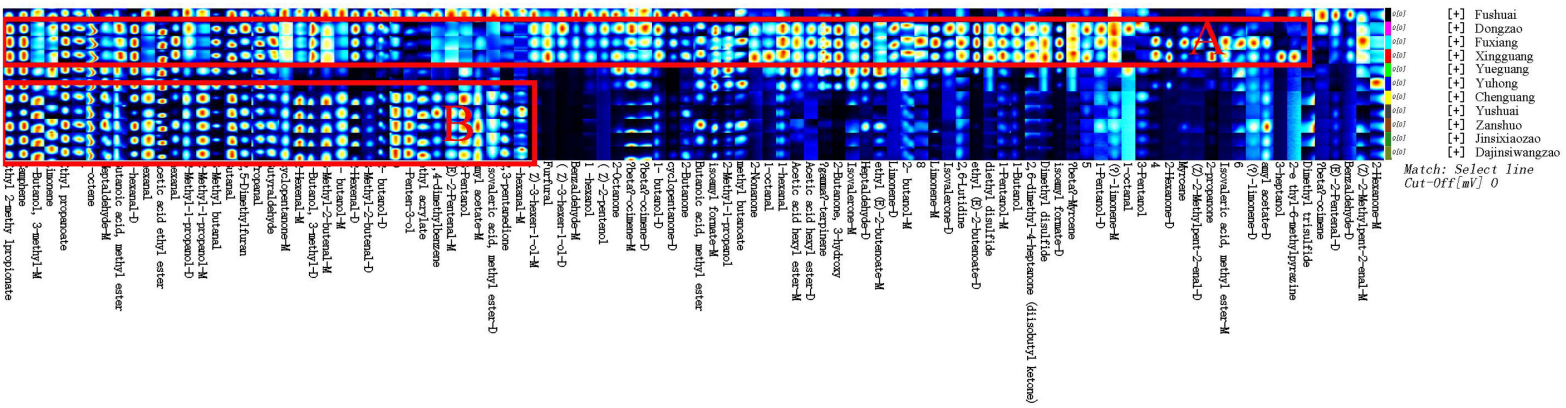
Volatile differences of different varieties of jujube flowers.

Based on the analysis of aroma profiles in jujube flowers across different growth stages, the flowering period was identified as the phase with the highest concentration of aromatic compounds. To identify cultivars with the most abundant aromatic profiles, we conducted a comparative study of 11 major jujube varieties during their flowering stage, representing nearly all principal cultivars currently cultivated. The analysis revealed that Fuxiang, Dongzao, and Xingguang exhibited the richest composition of aromatic compounds during flowering (**Figure 7**, red box A), whereas Yushuai, Zanshuo, Jinsixiaozao, and Dajinsiwang showed the lowest concentrations of these compounds (**Figure 7**, red box B). These findings suggest that Fuxiang, Dongzao, and Xingguang represent the most suitable varieties for the extraction of aromatic compounds from jujube flowers.

### 3.3 Differences in volatile flavor components between jujube flowers and other plant flowers

#### 3.3.1 Qualitative analysis of jujube flowers and other plant flowers in volatile flavor components

**Table 2** presents the qualitative analysis of volatile components in different plant flowers. A total of 102 signal peaks were detected, with 61 compounds successfully identified. These compounds were categorized as follows: 10 alcohols, 12 esters, 9 olefins, 11 aldehydes, 11 ketones, and 8 other substances, including 2 furans, 2 pyrazines, 1 aromatic hydrocarbon, 1 alkane, 1 acid, 1 amine and 8 substances unidentified.

**Table 2:** VOCs information of jujube flowers and other plant flowers detected by HS-GC-IMS.

Count	Compound	Flavor description	CAS#	Formula	MW^a^	RI^b^	Rt [sec]^c^	Dt [a.u]^d^	Comment
Alcohols									
1	(Z)-3-hexen-1-ol	woody, green	C928961	C6H12O	100.2	1408.5	970.643	1.24305	Monomer
2	(Z)-3-hexen-1-ol		C928961	C6H12O	100.2	1408.1	969.771	1.50963	Dimer
3	1-pentanol-4-methyl	fruity, green, nutty	C626891	C6H14O	102.2	1374.5	907.58	1.32286	Monomer
4	1-pentanol-4-methyl		C626891	C6H14O	102.2	1372.9	904.824	1.63624	Dimer
5	(E)-3-hexen-1-ol	green, cherry	C928972	C6H12O	100.2	1434.1	1020.711	1.24887	Monomer
6	(E)-3-hexen-1-ol		C928972	C6H12O	100.2	1432.5	1017.547	1.50653	Dimer
7	3-Pentanol	sweet, nutty	C584021	C5H12O	88.1	1107.3	478.13	1.40947	Monomer
8	3-Pentanol		C584021	C5H12O	88.1	1110.5	483.574	1.2089	Dimer
9	2-Methyl-1-propanol	pine, woody, camphor	C78831	C4H10O	74.1	1095.2	458.281	1.17063	Monomer
10	2-Methyl-1-propanol		C78831	C4H10O	74.1	1096	459.488	1.37312	Dimer
11	1-Butanol, 3-methyl	tea, nutty	C123513	C5H12O	88.1	1213.2	660.507	1.24244	Monomer
12	1-Butanol, 3-methyl		C123513	C5H12O	88.1	1213.3	660.744	1.48814	Dimer
13	Butanol	fruity, green, sweet	C71363	C4H10O	74.1	1142.1	540.484	1.18678	Monomer
14	Butanol		C71363	C4H10O	74.1	1141.1	538.505	1.39325	Dimer
15	1-Hexanol	fruity, green, sweet	C111273	C6H14O	102.2	1433.3	1019.129	1.31693	
16	1-Propanol, 2-methyl-	musty, pungent	C78831	C4H10O	74.1	1084.8	442.59	1.38605	
17	1-Penten-3-ol	fruity, green,	C616251	C5H10O	86.1	1159.6	574.631	0.93902	
Esters									
18	(E)-Ethyl-2-hexenoate	fruity, vegetable	C27829727	C8H14O2	142.2	1328.8	829.473	1.33273	Monomer
19	(E)-Ethyl-2-hexenoate		C27829727	C8H14O2	142.2	1331.1	833.232	1.81108	Dimer
20	isoamyl formate	wine, fruity	C110452	C6H12O2	116.2	1040.6	389.485	1.25895	Monomer
21	isoamyl formate		C110452	C6H12O2	116.2	1049.9	400.046	1.64025	Dimer
22	Methyl butyrate	fruity, green	C623427	C5H10O2	102.1	999.9	346.239	1.14681	Monomer
23	Methyl butyrate		C623427	C5H10O2	102.1	994.6	340.899	1.43606	Dimer
24	butyl 2-methylbutanoate	fruity, green, woody	C15706737	C9H18O2	158.2	1283.3	758.374	1.38831	Monomer
25	butyl 2-methylbutanoate		C15706737	C9H18O2	158.2	1282.2	756.772	1.8953	Dimer
26	Linalyl acetate	orange, lemon and pear	C115957	C12H20O2	196.3	1503.4	1170.136	1.2167	
27	Isobutyl 3-methylbutyrate	sweet, fruity	C589593	C9H18O2	158.2	1159.3	574.136	1.38587	
28	Ethyl 2-methy lpropionate	sweet, floral	C97621	C6H12O2	116.2	967.6	317.891	1.56062	
29	Butanoicacid propyl ester	fruity, green	C105668	C7H14O2	130.2	1142.1	540.484	1.27379	
30	2-methylbutanoic acid ethyl ester	apple, green	C7452791	C7H14O2	130.2	1025.4	372.67	1.6685	
31	Acetic acid ethyl ester	fruity, nutty	C141786	C4H8O2	88.1	898	267.419	1.33822	
32	Isovaleric acid, methyl ester	fruity, celery	C556241	C6H12O2	116.2	1023.1	370.268	1.52645	
33	Citronellyl formate	lemon, cucumber, scented, rose	C105851	C11H20O2	184.3	1569.9	1333.925	1.35067	
Alkenes									
34	myrcene	peach, fruity, green	C123353	C10H16	136.2	1193.7	635.631	1.15676	Monomer
35	myrcene		C123353	C10H16	136.2	1185.3	625.207	1.2878	Dimer
36	myrcene		C123353	C10H16	136.2	1187.6	628.05	1.59271	Trimer
37	Pinene	woody, camphor	C127913	C10H16	136.2	1159.6	574.631	1.21332	Monomer
38	Pinene		C127913	C10H16	136.2	1159.8	575.125	1.29296	Dimer
39	Pinene		C127913	C10H16	136.2	1158.3	572.156	1.71032	Trimer
40	3-Carene	orange, fruity	C13466789	C10H16	136.2	1184.7	624.496	1.66957	Monomer
41	3-Carene		C13466789	C10H16	136.2	1182.4	621.684	1.72612	Dimer
42	3-carene		C13466789	C10H16	136.2	1191	632.32	1.64607	Trimer
43	Terpinolene	sweet, floral, plastic	C586629	C10H16	136.2	1306.1	793.201	1.20996	Monomer
44	Terpinolene		C586629	C10H16	136.2	1306.6	794.001	1.32886	Dimer
45	Limonene	lemon, fruity	C138863	C10H16	136.2	1242.2	699.458	1.21107	Monomer
46	Limonene		C138863	C10H16	136.2	1242.2	699.458	1.28961	Dimer
47	Styrene	sweet, floral, plastic	C100425	C8H8	104.2	1193.1	634.92	1.51208	Monomer
48	Styrene		C100425	C8H8	104.2	1187	627.339	1.78171	Dimer
49	Camphene	camphor	C79925	C10H16	136.2	1061.9	414.227	1.21156	Monomer
50	Camphene		C79925	C10H16	136.2	1064.4	417.245	1.64671	Dimer
51	1-octene	oily, woody, lemon	C111660	C8H16	112.2	855.4	240.599	1.15205	
52	Dipentene	green, orange	C138863	C10H16	136.2	1250.1	710.413	1.63813	
Aldehydes									
53	2-Hexenal	almond, apple, plum	C505577	C6H10O	98.1	1230.9	683.961	1.17692	Monomer
54	2-Hexenal		C505577	C6H10O	98.1	1228.2	680.407	1.51334	Dimer
55	2-methyl-2-propenal	fruity, green, sweet	C78853	C4H6O	70.1	875.1	252.636	1.05846	Monomer
56	2-methyl-2-propenal		C78853	C4H6O	70.1	896.4	266.363	1.22148	Dimer
57	Heptaldehyde	fruity, pineapple, sweet	C111717	C7H14O	114.2	1243.2	700.745	1.3408	Monomer
58	Heptaldehyde		C111717	C7H14O	114.2	1246.4	705.242	1.7144	Dimer
59	Butanal	fermented, bready	C123728	C4H8O	72.1	848.3	236.375	1.1138	Monomer
60	Butanal		C123728	C4H8O	72.1	843.2	233.418	1.28086	Dimer
61	3-Methyl-2-butenal	nutty, cherry	C107868	C5H8O	84.1	1205.3	650.319	1.35584	
62	3-methylbutanal	chocolate, peach	C590863	C5H10O	86.1	858.6	242.499	1.19029	
63	Propanal	nutty	C123386	C3H6O	58.1	833.3	227.716	1.14198	
64	Butyraldehyde	bready, yeasty	C123728	C4H8O	72.1	912.1	276.922	1.2879	
65	Hexanal	green, woody, vegetative	C66251	C6H12O	100.2	1162.2	580.074	1.55547	
66	acrolein	butter, creamy	C107028	C3H4O	56.1	821.1	220.943	0.95984	
67	beta-ocimene	green, woody	C13877913	C10H16	136.2	1266.5	733.681	1.24493	
Ketones									
68	3-Hydroxy-2-butanone	cream, sweet	C513860	C4H8O2	88.1	1295.2	776.388	1.07785	Monomer
69	3-Hydroxy-2-butanone		C513860	C4H8O2	88.1	1294.4	775.187	1.33051	Dimer
70	2-Nonanone	fruity, oils, herbs	C821556	C9H18O	142.2	1326.2	825.226	1.39327	Monomer
71	2-Nonanone		C821556	C9H18O	142.2	1325.2	823.624	1.87879	Dimer
72	3-methyl-2-pentanone	fruity, pineapple, sweet	C565617	C6H12O	100.2	1016.6	363.343	1.17661	Monomer
73	3-methyl-2-pentanone		C565617	C6H12O	100.2	1023.2	370.288	1.47133	Dimer
74	3-Octanone	fruity, sweet	C106683	C8H16O	128.2	1205.3	650.319	1.30166	Monomer
75	3-Octanone		C106683	C8H16O	128.2	1206	651.267	1.71493	Dimer
76	2,3-pentadione	spicy, butter, creamy	C600146	C5H8O2	100.1	1024.8	372.034	1.31433	
77	Isovalerone	fruity, green, sweet	C108838	C9H18O	142.2	1243.7	701.52	1.78996	
78	3-Pentanone	minty, herbal, eucalyptus	C96220	C5H10O	86.1	984.3	331.288	1.35213	
79	2-propanone	sweet, green	C67641	C3H6O	58.1	815.9	218.102	1.12149	
80	Phellandrene	terpenes, spicy	C99832	C10H16	136.2	1132.1	521.679	1.67935	
81	2,3-Pentanedione	spicy, butter, creamy	C600146	C5H8O2	100.1	1102.8	470.707	1.30181	
82	Isomenthone	musty, herbal	C491076	C10H18O	154.3	1516.8	1201.261	1.34069	
Others									
83	methyl 2-furoate	fruity, mushroom	C611132	C6H6O3	126.1	1598.2	1410.462	1.15399	
84	Linalool oxide	green, sweet	C60047178	C10H18O2	170.3	1538.3	1253.306	1.2623	Monomer
85	Linalool oxide		C60047178	C10H18O2	170.3	1528.3	1228.814	1.81103	Dimer
86	3-Isopropyl-2-methoxypyrazine	green beans	C25773404	C8H12N2O	152.2	1478	1112.867	1.2397	
87	2,3-dimethyl-5-ethylpyrazine	fruity, pineapple, sweet	C15707343	C8H12N2	136.2	1538.3	1253.306	1.2281	
88	p-Cymene	fruity, sweet	C99876	C10H14	134.2	1265.3	731.953	1.20831	Monomer
89	p-Cymene		C99876	C10H14	134.2	1262.5	727.95	1.30574	Dimer
90	2,2,4,6,6-Pentamethylheptane	sweet, almond, fruity, green	C13475826	C12H26	170.3	918.5	281.357	1.31608	Monomer
91	2,2,4,6,6-Pentamethylheptane		C13475826	C12H26	170.3	926.3	286.847	1.40061	Dimer
92	2-Methylbutanoic acid	nutty, grassy	C116530	C5H10O2	102.1	1575.1	1347.701	1.20672	Monomer
93	2-Methylbutanoic acid		C116530	C5H10O2	102.1	1576.9	1352.294	1.44901	Dimer
94	Dibutylamine	wintergreen, almond, cherry	C111922	C8H19N	129.2	1120.8	501.389	1.74129	
Unknowns									
95	1		unidentified	*	*	1607	1434.954	1.33072	
96	2		unidentified	*	*	1131	519.699	1.54072	
97	3		unidentified	*	*	1106.5	476.895	1.46969	
98	4		unidentified	*	*	1046.5	396.123	1.45283	
99	5		unidentified	*	*	990.3	336.76	1.38932	
100	6		unidentified	*	*	962.8	314.09	1.50326	
101	7		unidentified	*	*	817.9	219.168	1.08806	
102	8		unidentified	*	*	787.9	203.461	1.13843	

a Represents the molecular mass.

b Represents the retention index calculated using n-ketones C4–C8 as external standard on MXT-WAX column.

c Represents the retention time in the capillary GC column.

d Represents the drift time in the drift tube.

#### 3.3.2 Analysis of volatile flavor components of different plant flowers

**Figure 8:**
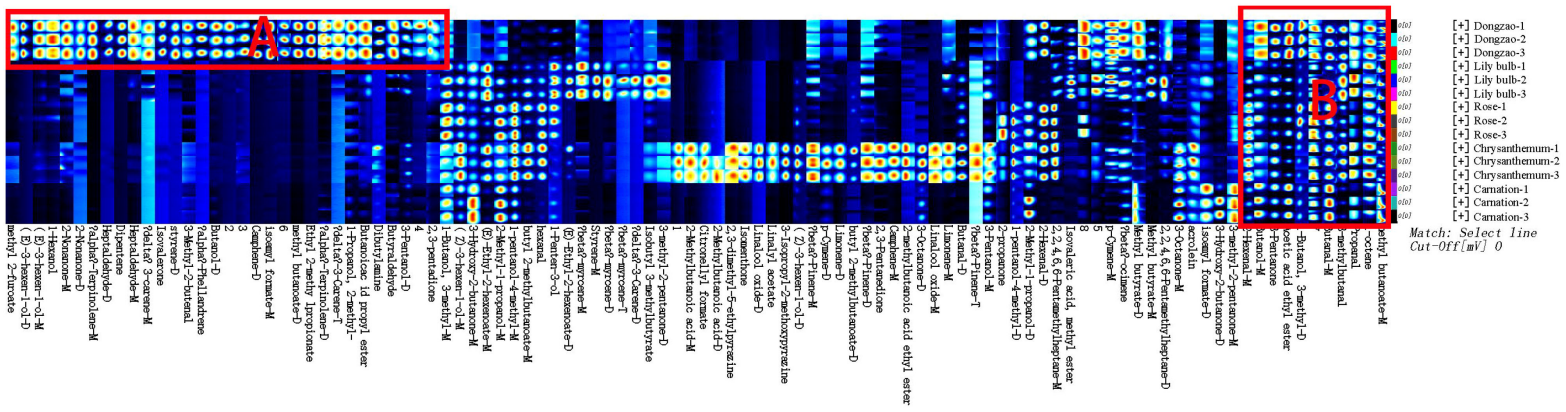
Comparison of HS-GC-IMS fingerprints of volatile organic compounds (VOCs) in jujube flowers and other plant flowers samples.

**Figure 9:**
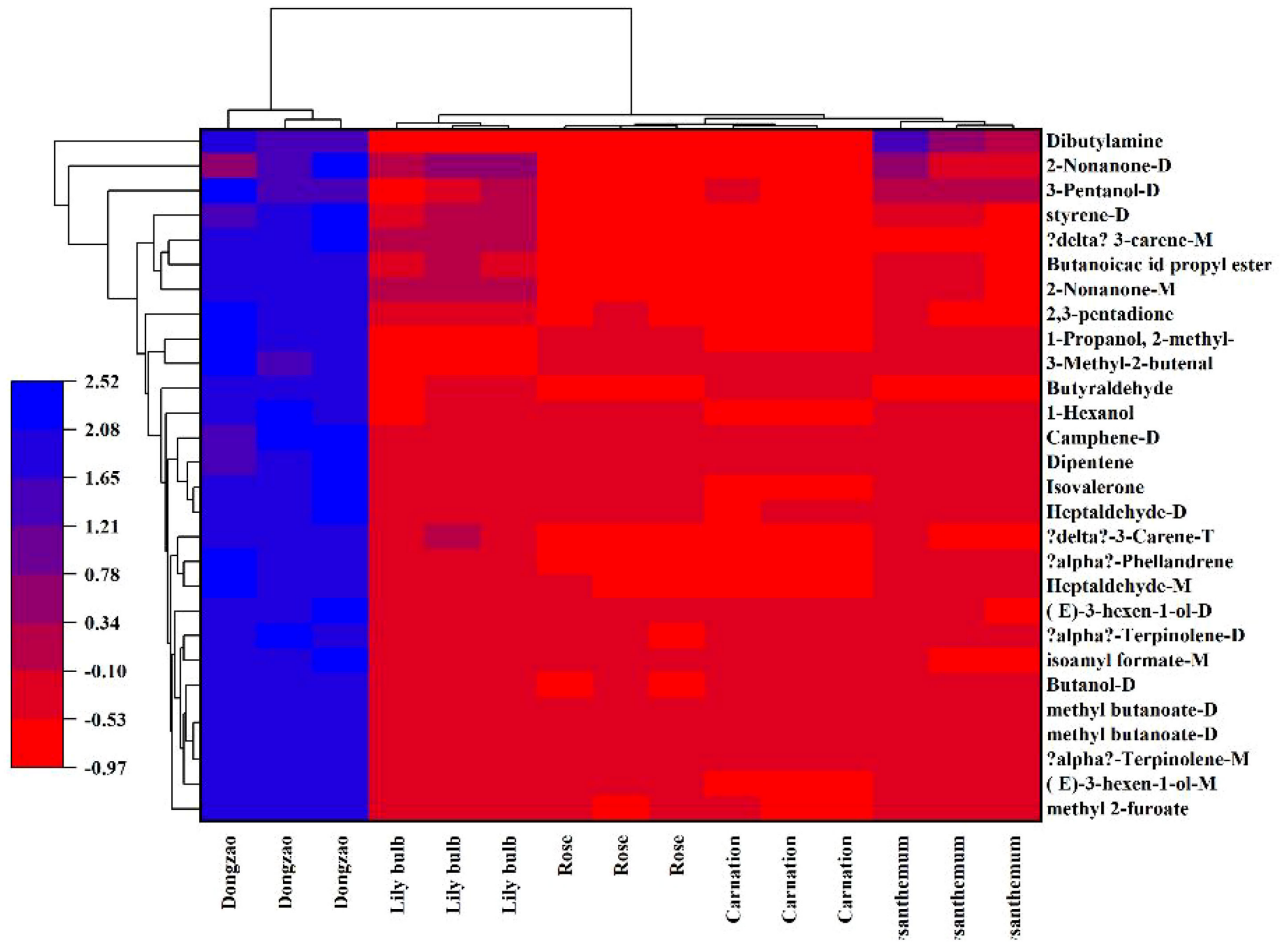
Screening of differential volatile components in jujube flowers.

As shown in **Figure 8**, the aroma profile of jujube flowers exhibits significant differences compared to the other four plant species. Common volatile compounds detected across all plant flower samples include methyl butyrate, 1-octene, butanol, acetic acid ethyl ester, 3-methyl-2-butenal, 3-pentanol, propanal, 2-hexenal, and 1-butanol, 3-methyl (**Figure 9**, red box B). Notably, jujube flowers contain 24 unique aroma components and 4 unidentified compounds (**Figure 8**, red box A). The clustering heat map analysis further confirmed that these 24 volatile compounds effectively distinguish jujube flowers from other samples (**Figure 9**).

Several key compounds contribute to the distinctive aroma and functional properties of jujube flowers. For instance, (E)-3-hexen-1-ol, 3-pentanol, 1-hexanol, 1-propanol, 2-methyl-, (E)-ethyl-2-hexenoate, isoamyl formate, isobutyl 3-methylbutyrate, butanoic acid propyl ester, phellandrene, 2,3-pentadione, 2-nonanone, heptaldehyde, and dipentene impart fruity, nutty, and creamy aromas to jujube flowers. Additionally, terpinolene and α-phellandrene exhibit anti-inflammatory and antioxidant activities, which are beneficial for *in vitro* wound healing (de Christo Scherer et al., 2019). Camphene, commonly found in vegetables and herbs (Tiwari and Kakkar, 2009), is also present in jujube flowers. Isoamyl formate, a colorless oily liquid with a distinctive plum and black currant aroma, possesses antimicrobial properties that inhibit or kill bacteria, yeast, filamentous fungi, and oomycetes, thereby promoting plant health (Hummadi et al., 2022). These compounds collectively protect jujube flowers from pests and diseases during their development. Ketones, which are key products of the Maillard reaction and precursors to many flavor compounds (Smuda and Glomb, 2013),are also prominent in jujube flowers. For example, 2-nonanone, a colorless to light yellow liquid with an herbal aroma, can be utilized in active packaging systems to preserve fruit freshness post-harvest and prevent flavor degradation caused by high volatile content (Almenar et al., 2009). Terpinolene, 2-nonanone, 2,3-pentadione, and phellandrene are major components of the yellow liquid in jujube nectar. Furthermore, 3-carene, 3-pentanol, and isoamyl formate in jujube flowers contribute to pathogen resistance, underscoring the dual medicinal and nutritional value of jujube fruit (Shu et al., 2020; Song et al., 2015).These findings highlight the unique aromatic and functional characteristics of jujube flowers, which are integral to their role in both ecological and agricultural contexts.

#### 3.3.3 Jujube flowers and other plant flowers PCA of volatile flavor components

**Figure 10:**
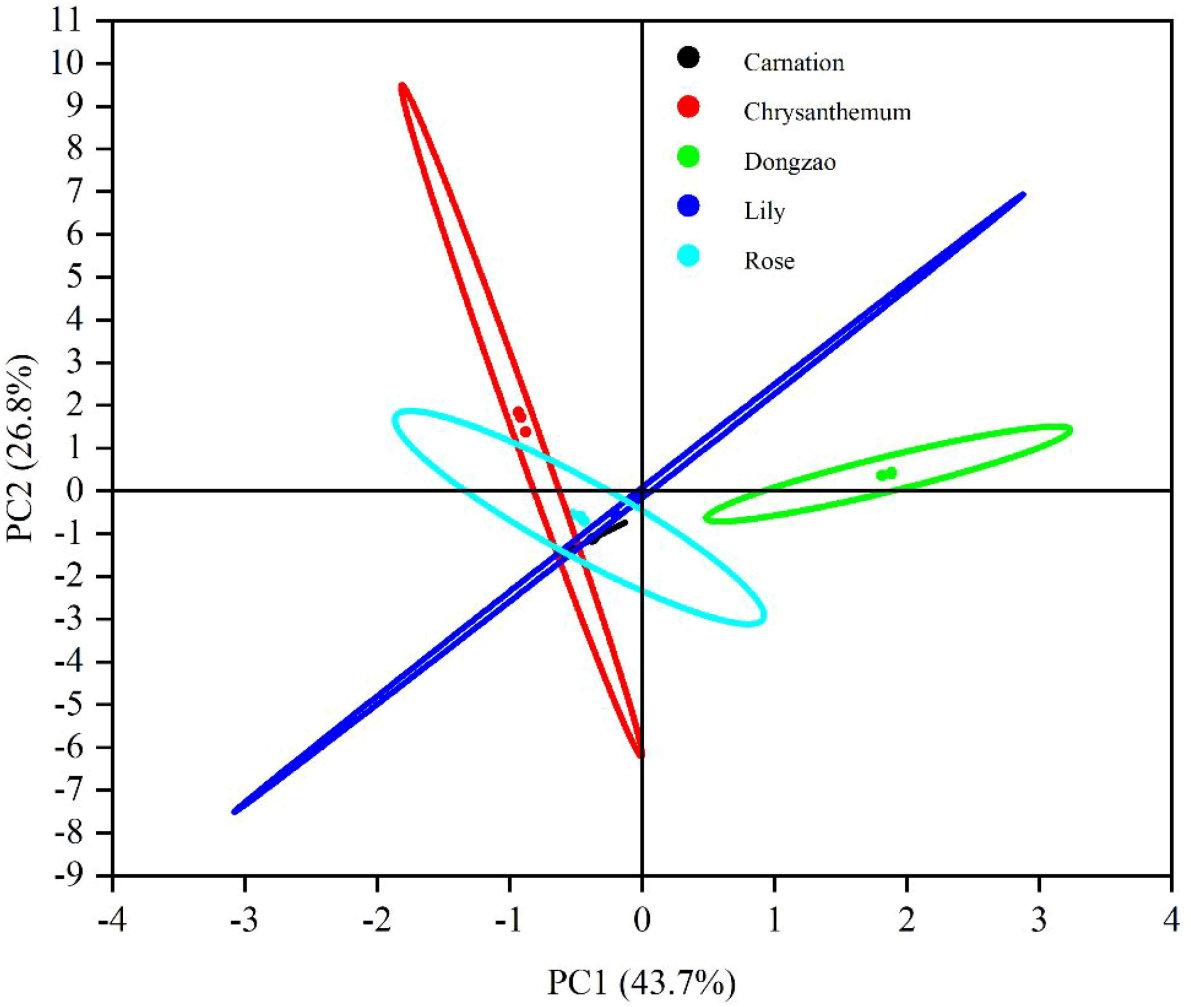
PCA analysis results of volatile components of jujube flowers and other plant flowers.

Principal Component Analysis (PCA) of the fragrance profiles of jujube flowers and four other plant species revealed a total contribution rate of 70.5% for the principal components, with PC1 accounting for 43.7% and PC2 for 26.8% of the cumulative variance. This level of contribution is sufficient to effectively capture the differences in fragrance between jujube flowers and the other plant species (**Figure 10**). The significant spatial separation observed between jujube flowers and the other four plant species in the PCA plot further underscores the distinctiveness of jujube flower fragrance. These findings align well with the results presented in the fingerprint analysis (**Figure 8**), reinforcing the conclusion that jujube flowers possess a unique aromatic profile compared to the other plants studied.

#### 3.3.4 Similarity analysis of 24 characteristic volatile organic compounds of jujube flowers

**Figure 11:**
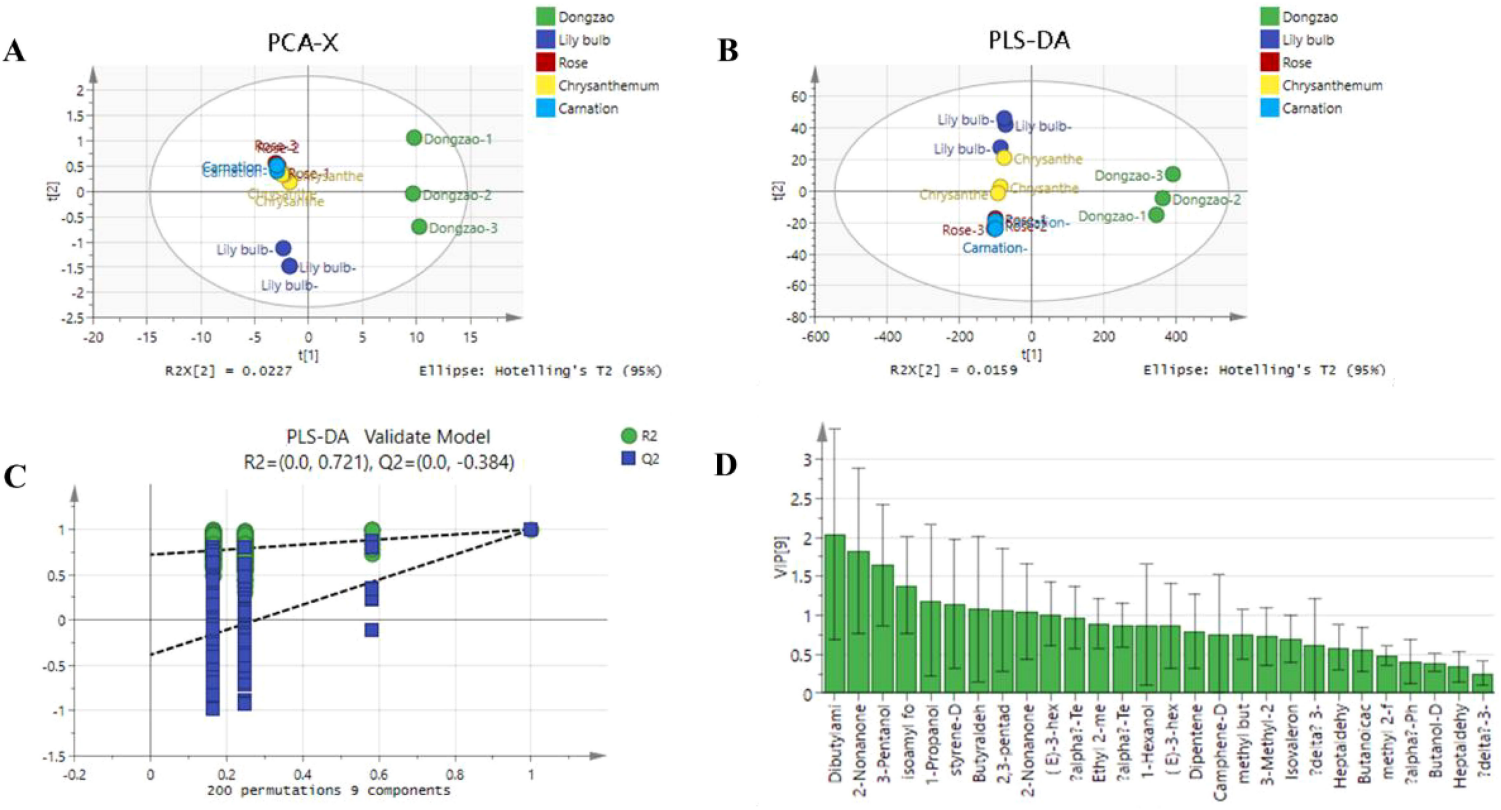
PCA analysis of 24 characteristic aroma compounds of jujube flowers and other plant species. **(A)** PCA-X score plot showing clustering of Dongzao, Lily bulb, Rose, Chrysanthemum, and Carnation samples. **(B)** PLS- DA score plot with similar groupings. **(C)** PLS-DA validation model displaying R2 and Q2values across permutations. **(D)** Bar chart of variable importance in projection (VIP) scores for different chemical compounds, with Dibutylam and 2-Nonanone as top compounds.

A PCA model was constructed to analyze the 24 characteristic aroma compounds of jujube flowers and other plant species. The results revealed that jujube flowers were predominantly located in the first and fourth quadrants, demonstrating a clear distinction from the other four plant species (**Figure 11**). In this study, the model exhibited excellent fit and predictability, as indicated by R2X, R2Y, and Q2 values approaching 1. **Figure 11** further illustrates that the aroma profile of jujube flowers is distributed across the first and fourth quadrants, consistent with the PCA results. To ensure the robustness of the model and avoid overfitting, a permutation test was conducted. As shown in **Figure 11**, after 200 cross-validations, the Q and abscissa intercepts were negative, and the R2 and Q2 values remained below the original values, confirming that the model did not overfit the data.

The contribution of individual aroma compounds to the overall profile of jujube flowers was evaluated using VIP (Variable Importance in Projection) values. As depicted in **Figure 11**, nine aroma components with VIP values greater than 1 were identified as significant contributors: dibutylamine, 2-nonanone, 3-pentanol, isoamyl formate, 1-propanol, 2-methyl-, styrene, butyraldehyde, 2,3-pentadione, and (E)-3-hexen-1-ol. These compounds are considered the primary volatile substances responsible for the distinctive aroma of jujube flowers.

### 3.4 Sensory evaluation of jujube flowers and other plant flowers volatile flavor components

**Figure 12:**
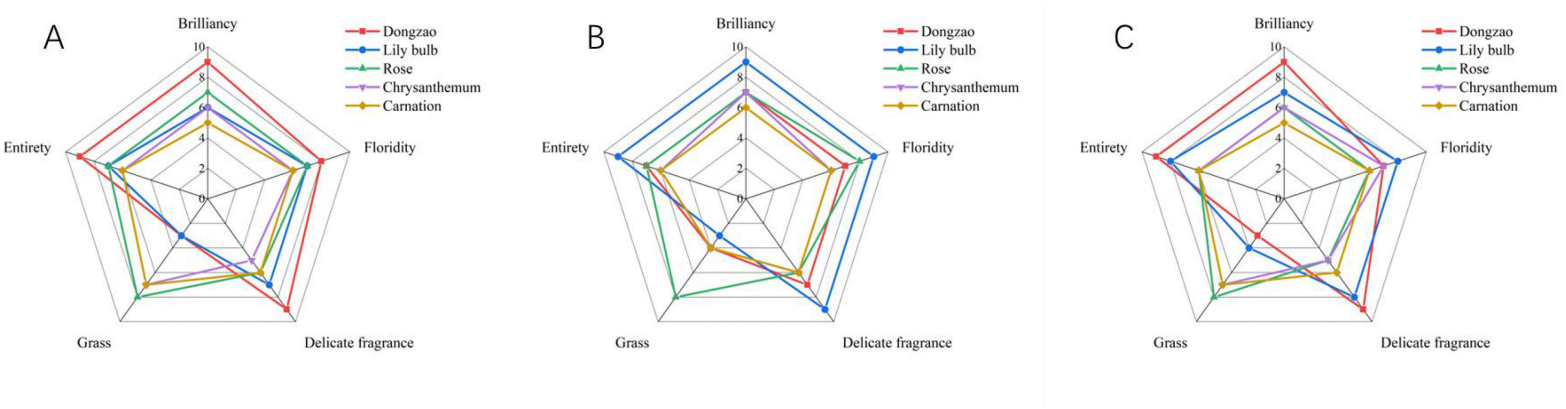
The sensory evaluation map of different plant floral fragrances. **(A)** Men’s sensory evaluation score. **(B)** Women’s sensory evaluation score. **(C)** Comprehensive sensory evaluation score.

For sensory evaluation, we recruited a panel of 10 male and 10 female participants, which revealed significant gender-based differences in fragrance preferences. Most male participants expressed a preference for the fragrance of jujube flowers, describing it as pleasant and unique compared to the aromas of the other flowers (**Figure 12**). In contrast, female participants tended to favor more vibrant and fragrant flowers, with Lily bulb receiving the highest score among them (**Figure 12**). These results highlight the differing aromatic preferences between men and women, with jujube flowers and Lily bulb representing the favored choices of each group, respectively (**Figure 12**).

Based on the positive feedback from male evaluators regarding the fragrance of jujube flowers (Dongzao), there is potential to extract and utilize its pleasant aroma for the development of male-oriented fragrances or air fresheners in the future. This approach aligns with the sensory preferences identified in the study and offers a promising avenue for product innovation.

## 4 Conclusion

Aroma is a critical factor in evaluating jujube flowers. In this study, we employed GC-IMS combined with fingerprint analysis and PCA to identify the primary aroma components in jujube flowers. The results revealed that the flowering stage contains the highest concentration of aroma components, with 14 specific compounds showing a significant increase from the bud stage to the flowering stage. Further analysis using clustering heat maps and PLS-DA highlighted 24 characteristic aroma components in jujube flowers, among which 9 were identified as the dominant contributors to their fragrance. Dongzao, Fuxiang, and Xingguang were identified as the three jujube varieties with the most abundant and distinctive aromatic profiles. These findings confirm that GC-IMS combined with fingerprint and PCA analysis is an efficient and reliable method for studying jujube flower aromas. Additionally, sensory evaluation revealed that male participants exhibited a stronger preference for the fragrance of jujube flowers, describing it as pleasant and appealing. This suggests potential applications for jujube flower aroma in the development of men’s perfumes, air fresheners, aromatherapy products, and more. In conclusion, the analytical methods employed in this study provide valuable insights into the flavor substances of jujube flowers and offer a foundation for further research and product development.

## Data Availability

The original contributions presented in the study are included in the article/**Supplementary Material**, further inquiries can be directed to the corresponding author/s.
